# A Few More Cases Cured with the High and Highest Potencies*See The Homœopathic Physician for August, 1892, page 364.

**Published:** 1893-05

**Authors:** J. Emmons

**Affiliations:** Richmond, Ind.


### A FEW MORE CASES CURED WITH THE HIGH AND HIGHEST POTENCIES.*

J. Emmons, M. D., Richmond, Ind.

Case I.—Was called to a neighboring village six miles distant to see Mrs. B——, supposed to be near death's door from facial phlegmonous erysipelas. Face swollen beyond recognition. It had commenced and was still worse on the left side—purple, with burning pain. She would awaken from short sleep in great distress, and was afraid to go to sleep again. She could not allow anything around the neck or even touch it. Sense of suffocation. She could hardly get her breath at times from a choked feeling. Lach.$^{cc}$, three doses three hours apart. Much better in twenty-four hours, and made a good recovery without more medicine.

Case II.—Mrs. R——, whilst still in bed, one week after

* See The Homœopathic Physician for August, 1892, page 364.

confinement, was attacked with facial erysipelas. In twelve hours the left eye was closed, color purple and burned like fire. Placed one dose of Lach.$^{cc}$ on the tongue; called the next morning. Swelling nearly gone; eye open—and when asked about the burning, said she "had felt nothing of it since I placed that powder on her tongue," that was the last of it.

CASE III.—Mr. S——, age twenty-four; single; typhoid fever. At the end of second week his father came to my office at six o'clock of one of the coldest mornings last winter. Said John had bloody stool and was much worse. I gave him two powders of Alumina$^{1000}$, told him to give one on his return and the other in one hour, and I would see him on going out at nine o'clock. Before the father's return the patient had a second stool, the two filling a wooden bucket two-thirds full—they had kept them for my inspection. At my visit found him cold and pulseless, but as there had been no hemorrhage since taking the first powder (unless it was concealed), I concluded to await results, and would call again in the evening. At my second visit I found reaction had taken place. No more hemorrhage, and he, too, made a good recovery without another dose of medicine.

CASE IV.—Mr. R——; married; about twenty-five years old. He too lived six miles away, had been sick some time from a mismanaged or neglected pneumonia. Cough, night-sweats, frequent pulse, and, to all appearances, as if in the last stage of pneumonic-consumption. An abscess had burst that day and was discharging a great amount of pus. He said it "tasted like *matter*." He received three doses of Pyrogen$^{cm}$ that day, three hours apart. Made a rapid recovery without any more medicine.

